# 3D Scanning of Wood–Plastic Composite Decking After Cyclic Thermal Action

**DOI:** 10.3390/ma18010097

**Published:** 2024-12-29

**Authors:** Artur Piekarczuk, Ewa Szewczak, Ewelina Kozikowska, Łukasz Gołębiowski

**Affiliations:** Instytut Techniki Budowlanej, 00-611 Warsaw, Poland; e.szewczak@itb.pl (E.S.); e.kozikowska@itb.pl (E.K.); l.golebiowski@itb.pl (Ł.G.)

**Keywords:** construction products, wood–plastic composites, 3D scanning, SEM test, test method uncertainty, plane deformations

## Abstract

Wood–plastic composites (WPC) combine the properties of polymers and wood, providing an attractive alternative to traditional materials, particularly for terrace flooring. When exposed to various environmental conditions, WPCs are affected by factors, such as water and ultraviolet (UV) radiation. Although most test methods for assessing the durability of these products have focused on changes in mechanical properties and linear dimensions, out-of-plane deformations (concavity and convexity) are often overlooked. This study focusses on evaluating the usefulness of the test method that allows for precise determination of these deformations after ageing. The test procedure involves exposure to classic weathering for decking boards, including moisture, UV radiation, and water spray, followed by three-dimensional (3D) scanning to track deformation after different exposure times. Analysis of variance was used to assess whether the sensitivity of this method is sufficient to detect minor deformations. Additionally, scanning electron microstructural images of the aged samples were examined to determine whether there was a relationship between the deformation and the microstructural changes. This study demonstrated the potential to use scanning methods for assessing the aspects of ageing resistance of this type of composite product in the context of deformation.

## 1. Introduction

Environmental concerns and the recycling of construction materials have led to great interest in polymeric materials with natural fillers and their properties, and the literature in this field is quite rich, for example, [1,2,3,4]. According to the bcc Research report [5], the global market for biobased composite materials is estimated to be worth USD 5.9 billion in 2023 and will grow to USD 9.0 billion by 2029. Wood–polymer composites (WPC) [6,7,8] are an excellent solution for those looking for a material that has properties similar to wood but has better water resistance, can be economically processed into a final product, and has satisfactory properties stability in many environments. One application of such composites is in the form of prefabricated planks to cover floors on outdoor terraces [9]. Prefabricated products, that is, floorboards, are exposed to a wide range of environmental conditions, and their durability is determined by their service life and intensity of exposure to the elements. Floorboards are usually in the form of a chambered profile several tens of millimetres wide with air voids inside the shape of the chambers. This is a relatively simple shape, which should provide sufficient rigidity and at the same time have a relatively low dead weight. The structural structure of the pre-cast is considerably more varied. In a WPC composite, the matrix is a polymer (usually polyethene, polypropylene, or polyvinyl chloride [4]), and the filler is wood in various forms, such as shavings, sawdust, splinters, wood chips, or, most commonly, wood flour. Matrix selection depends on the intended use. Civil engineering applications are dominated by WPC with polyvinyl chloride (PVC) or high-density polyethylene (HDPE) matrix [4]. They are used in solid or cellular profiles intended for outdoor floors, such as terraces and swimming pools. Because of the unique combination of polymer and wood properties as well as their attractive appearance, WPC composites have become an interesting alternative to polymers and products in the wood industry. Wood flour is a material that is typically produced by mechanically grinding wood in several stages in rotary hammer-type shredders. It has lipo- and hydrophilic properties and is insoluble in water. Depending on the amount of wood flour introduced into the polymer, the adsorption properties of the composite produced can be modified [10]. The composition of wood moss depends on the species of the tree, while its purity depends on the condition and region of origin, as well as the process from which the waste is produced [11].

The WPC composite profiles were manufactured by extrusion or coextrusion. In this technology, profiles are extruded from thermoplastic materials that change their aggregation state from solid to flexible under the influence of heat and pressure. Extrusion is a profile-forming process that uses a single mixture of raw materials. The mixture for the WPC composite, consisting of wood flour and polymers, was pushed through a moulding die, which gave the desired shape to the profile. Thus, the final product consisted entirely of a single raw material. Coextrusion, on the other hand, is a method of multilayer manufacturing of composite profiles, in which two blends of raw materials are used, which are combined during a single production process, allowing better strength, aesthetics, or functional performance.

The tests to which terrace decking profiles made of wood–polymer composites are subjected before they are placed on the market derive primarily from the EN 15534-4 standard [12] on decking profiles and tiles for external use, made from wood–polymer composites or natural fibre composites (NFC), and the European Assessment Document (EAD) 190005-00-0402 [13] on terrace decking kits. The two documents, which are partially interrelated, provide for tests of the mechanical and physical properties of products immediately after manufacture, as well as tests of resistance to external factors, including weathering. According to EN 15534-4, the resistance to weathering is determined by changes in colour and resistance to moisture by changes in flexural strength and water absorption. EAD also recommends testing for UV radiation, taking as an indicator change in Charpy impact strength. Linear deformation of the boards is determined only by swelling when exposed to moisture. Out-of-plane deformation changes (concavity and convexity) are not included as a durability parameter. On the basis of the authors’ own experience in this publication, this type of deformation can deteriorate the functional characteristics of floors. Furthermore, out-of-plane deformations can be indicative of structural degradation of the composite material itself, with major consequences for its durability.

Traditional measurement methods (e.g., using point sensors), especially for small out-of-plane deformations, are time-consuming and technically difficult to implement. The most effective method appears to be vision-based observation and, in particular, image acquisition using 3D scanning technology and image processing using inspection engineering methods [14,15]. Three-dimensional scanning technology has been developed for more than two decades and has been applied in various areas of technology. It is developing particularly rapidly in the precision industry for quality control of retail production, especially in precision quality control measurements. In general, scanning is the optical capture of information about the geometry of physical objects. Currently, the most commonly used scanning techniques use different types of projection units. These include laser, structured light, and infrared light scanners. Regardless of the technique used, each scanning process produced a numerical image that represented the topography of the object. The 3D images obtained can have different representation accuracies depending on the device used. For precision measurements, a measurement accuracy of ±0.01 mm is achieved. After the object, the information was digitally stored as a point cloud. The point cloud was then converted to STL surface meshes (standard triangle language) using appropriate graphics tools [16]. The numerical representation of the topography of the object completes the model acquisition stage and can be used in various survey scenarios. The simplest is that the scan is used to reconstruct the object documentation, that is, to build a computer-aided design (CAD) model. Reverse engineering has been used for this purpose [17,18]. The result of the reverse engineering implementation is the acquisition of a digital model, that is, a representation of the object’s geometry with features similar to the original. This model can be further processed as a batch file for numerical simulations using the finite element method, or it can form wider computer structures, such as a digital twin [19]. Other applications of models are control engineering [20] and inspection engineering [14]. The scanned model has all the geometrical features of the object, including its defects. Juxtaposing the scan with a 3D CAD design or another scan creates an inspection pair. Objects juxtaposed in this manner can be compared with each other by determining the shape deviations. It is precisely this application of scanning and inspection techniques that is included in the example analysed.

The 3D scanning process and inspection analysis were the only tools used to obtain test measurement results. Typical testing of building products involves many steps, including measurements, exposure to environmental impacts, and visual assessments. The suitability of a measurement or test method for a given application can be considered from two perspectives: the relationship to the conditions of use of the product and the metrological properties of the method, such as its accuracy. The aim of this study is related to the metrological aspect of test methods and is to assess whether the test method based on standard exposure of terrace boards to atmospheric factors and then measurement of their plane deformations (concavity and convexity) can be used, along with other methods, to assess the resistance of composite terrace boards to atmospheric factors.

The suitability of a test method should be based on its ability to reveal differences in the phenomena that occur or their effects. To some extent, the uncertainty value of the result contains information on the accuracy of the test method. This includes the resolution of the measurement itself and many other factors that influence the dispersion of the results. There are many discrepancies in the uncertainty estimation of the test results ([21,22,23]), including those related to its dependence on the material under study. Therefore, the value of the uncertainty estimated according to commonly used rules [24] alone may not give a clear answer to the usefulness of the method. In this paper, a variance analysis was used to assess the suitability of the 3D scanning method for observing the deformations that occur in building products after ageing. Composite planks with relatively small deformations during ageing were chosen for this study to determine whether, with small deformations, the method can capture differences in the deformation of individual points on the samples, differences due to the effect of exposure time, or differences between individual samples.

To confirm the sense of the out-of-plane deformation test as an indicator of durability (weather resistance), microstructure SEM (scanning electron microscope) testing was conducted to determine whether there was a relationship between the deformation and microstructural changes.

## 2. Materials and Methods

### 2.1. Test Sample and Climatic Impacts

**Figure 1:**
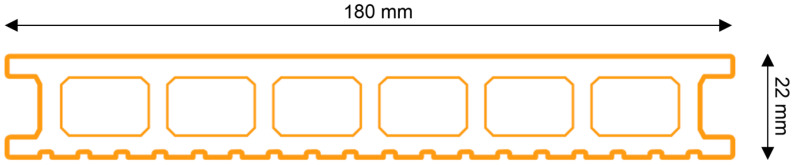
Sample cross-section.

The tests were carried out on the chamber profiles (Figure 1) for floor coverings on outdoor terraces. Five samples (AE) were taken for testing. The width and thickness of the profiles were 180 and 22 mm, respectively. The length of the samples was 300 mm. Profiles were made from NFPC (natural fibre-reinforced polymer composite) with a PVC matrix and a filler of fine lignocellulosic fibres in the form of the so-called wood flour, recycled from wood industry waste, with the addition of plasticisers and modifiers, including UV absorbers. The composition of the composite is a company secret that has not yet been disclosed. Profiles were produced using an extrusion process.

**Figure 2:**
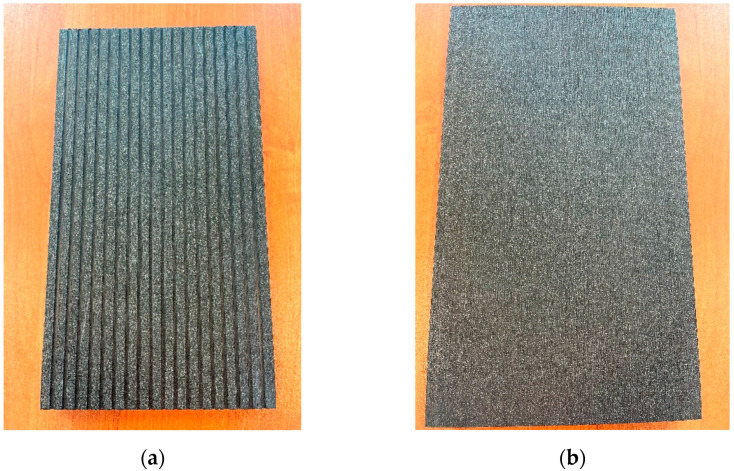
Useful surfaces of profiles (**a**) fluted (**b**) plane (brushed).

The profiles had two wear surfaces: fluted and plane (Figure 2). Typically, both surfaces are mechanically brushed with a metal brush to give them a wood-like appearance.

The ageing procedure was carried out using a UV test apparatus (Atlas, Linsengericht, Germany) equipped with type 1A fluorescent lamps (UVA-340), according to ISO 16474-3 [25], which emit light with a wavelength between 300 and 400 nm, with an emission maximum at 343 nm (Table 1). The exposure pattern was in accordance with EN 927-6 [26]. The samples were cycled, consisting of a long condensation phase followed by exposure to UV lamps at an irradiance of 0.89 W/m^2^ measured at 349 nm, alternating with wetting by water spray. Specimens cut from the flat surfaces of usable profiles from a brushed surface were exposed. At the time of exposure, the samples were placed at an angle of approximately 80 ° to allow the water to drain freely. Exposures lasting a total of 3024 h were applied. The course of exposure for each cycle is shown in Table 1.

### 2.2. 3D Scanning Method and Inspection Analysis

**Figure 3:**
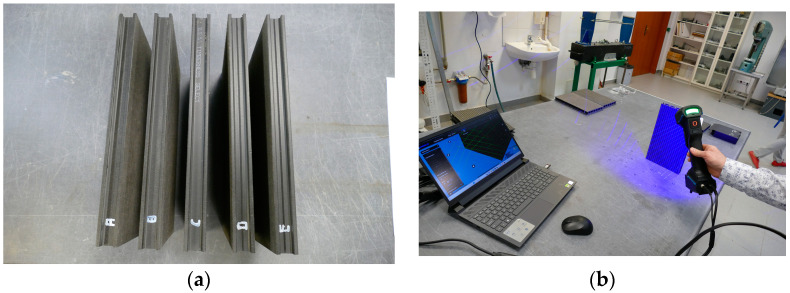
Scanning of test samples, (**a**) test samples, (**b**) scanning process, A–E: sample symbols.

Three-dimensional imaging was performed using a 3D handheld scanner FreeScan Trio 3D (SHINING 3D, Hangzhou, China (HQ)). Sample scanning was performed using a handheld laser scanner, FreeScan Trio 3D [27] (Figure 3). Basic scanner parameters: light source blue laser 26 cross lines; scanning accuracy to 0.02 mm; volumetric accuracy in photogrammetry mode: 0.02 mm + 0.015 mm/m; scanning speed 3,010,000 pct/s.

The scans were performed using several different sequences. The first was the reference scan, that is, before the climatic impact. The following scan sequences were operational scans, that is, after climatic impact. The reference and operational scans were compared with each other in the Geomagic Control X inspection programme (v. 2024.1). The comparison resulted in a quantitative and qualitative assessment of the deformation. The algorithm used to determine the deviations consists of calculating the deformation as a scalar quantity determined according to Relation (1).

(1) $$D=\sqrt{{GV}_{x}^{2}+{GV}_{x}^{2}+{GV}_{z}^{2\;}},$$

The *GV* vector is defined as the positional difference between a point in the STL mesh of the deformed operational model and its corresponding point on the undeformed reference model. The calculation of these vectors was based on an established reference system, specifically the normal to the reference surface. Reference points, in general, are determined as follows.

(2) $$P_{o}=\langle x_{0}\;{,y}_{0}\;{,z}_{0}\;\rangle \;;\;P_{r}=\langle x_{r}\;{,y}_{r}\;{,z}_{r}\;\rangle$$

where *x*, *y*, and *z* are the coordinates of the node indexes: *o*, operational model, *r*, reference model.

Vector:

(3) $$GV=\langle x_{o}-x_{r},\;y_{o}-y_{r}{,\;z}_{o}-z_{r}\rangle ,$$

The results of the inspection can be presented in two primary formats: quantitatively, as discrete points with precise deformation values, or qualitatively, as a deformation map with an indicative deformation scale. In the quantitative approach, the deformation is calculated according to Relation (1), where the result represents an estimate of the expected value in the immediate vicinity of the measurement point. It should be emphasised that this result is inherently associated with a standard uncertainty that arises from the data acquisition methodology.

When a result is determined for a specific point, it is calculated as the mean value of the STL mesh nodes located within a defined neighbourhood of the selected point. In the case of a deformation map, the standard deviation is determined based on points distributed throughout the observation area, allowing for the assessment of deformation patterns, including the geometric distribution and intensity of the extrema. For sectional analyses, the standard deviation of displacements is calculated specifically for nodes situated along the section line. This enables a detailed evaluation of the deformation within a given region of interest.

### 2.3. Statistical Analysis

The deformation of the samples was tested by measuring the deviations from the baseline dimensions in five samples at ten measurement points on each sample. Five measurement points were located on the plane surface and five on the fluted surface. To determine the feasibility of drawing conclusions about the behaviour of the samples based on the measurements, analysis of variance (ANOVA) tests were adopted as the primary method for analysing the statistical significance of differences between the results. ANOVA was preceded by the normality test. Although with small sets of results, there are no normality tests that provide fully reliable results, it was decided to conduct the Shapiro–Wilk normality test (with a 5% probability of error). This test was chosen because it is generally considered to have relatively high power (e.g., [28,29,30]).

Shapiro–Wilk tests were performed on sets of results, where the variable was:

(P) Point on the sample. Sets of results were obtained for testing, each for the same exposure time and the same sample. The sample points on a plane surface (1 < 5) and a fluted surface (6 < 10) were tested separately. A total of 42 sets of four to five results were tested.
(S) Sample. Sets of results for different samples were obtained for testing, but each for the same point and exposure times. Fifty sets of four to five results were tested.
(E) Exposure times. Sets of results for different exposure times were obtained for the test, but each for the same point and the same sample. A total of fifty sets of four to five results were tested.

A two-way analysis of variance was used to assess the significance of differences between sets of results to initially assess the influence of the following factors on the measured deformation:

(a) Effect of sample and exposure time for each point on the sample separately (ten sets of results, each at a different point on the sample).
(b) Effect of the point on the sample and exposure time for each sample separately. (ten sets of results: five for five samples for points 15 and five for points 6 ÷ 10).
(c) Effect of sample and point for each exposure time separately (ten sets of results: five for five exposure times for points 15 and five for points 6 ÷ 10).

One-way analysis of variance was performed for pairs of sets of results obtained for individual points on the samples, treating the points on the plane surface and the points on the fluted surface separately. The same analysis was performed for pairs of sets of results for two different exposure times to assess the influence of exposure time on the deformation.

### 2.4. Microstructure SEM Analysis

The microstructure of the coated surface was investigated using a Sigma 500 VP scanning electron microscope with cold-field emission (Carl Zeiss Microscopy GmbH, Köln, Germany).

The microstructure surfaces of the coatings were examined at an accelerating voltage of 5 keV excitation electron beam using an SE detector on samples sprayed with a coat of gold. SEM microscopic observations of the chamber profiles were made on the brushed surface in the initial state and after ageing for 18 cycles (3024 h). The samples for testing were cut from the corners and central area of the profiles. All observations were made at 200× and 1000× magnification.

## 3. Results

### 3.1. Measurement Inspection

**Figure 4:**
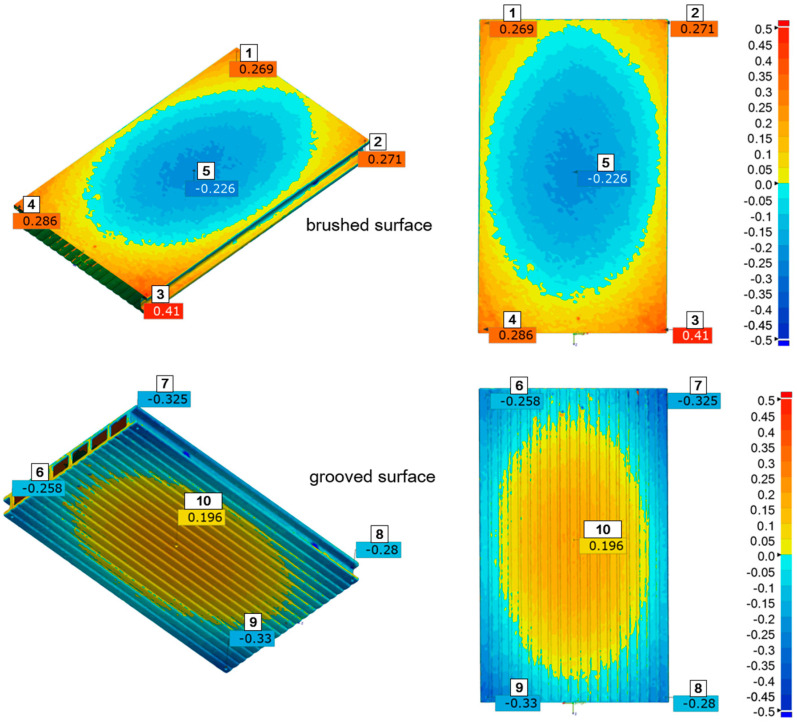
Deformation map of the specimen deformation after four ageing cycles with marked measurement points on the brushed and fluted surfaces. Deformations are expressed in micrometres, 1–10: measuring points.

**Figure 5:**
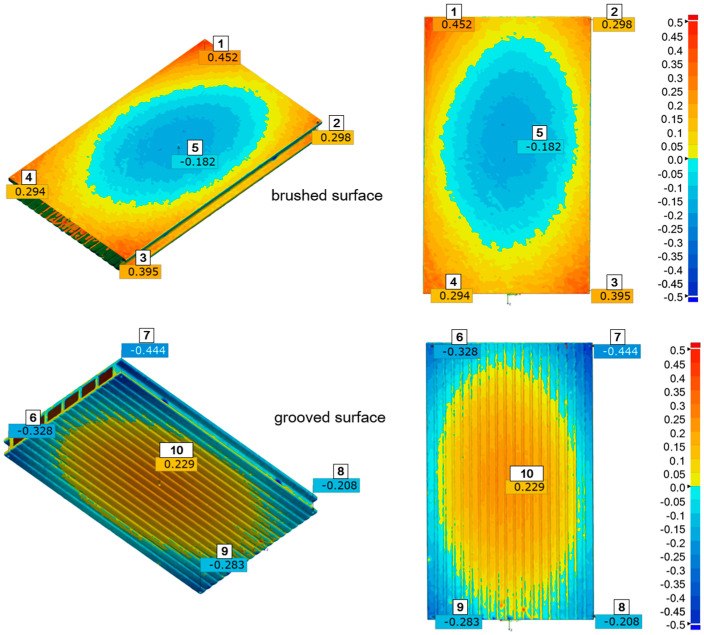
Deformation map of the specimen after eight ageing cycles with marked measurement points on the brushed and fluted surfaces. Deformations are expressed in micrometres, 1–10: measuring points.

**Figure 6:**
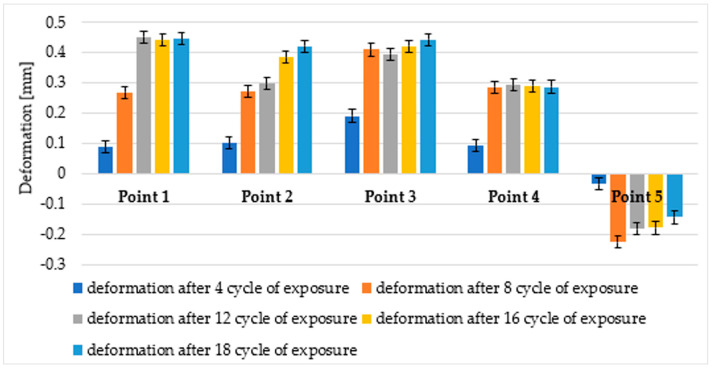
Deformation at measurement points on the brushed surface in successive ageing stages. The error bars show the expanded measurement uncertainty estimated as described in Section 3.4.

**Figure 7:**
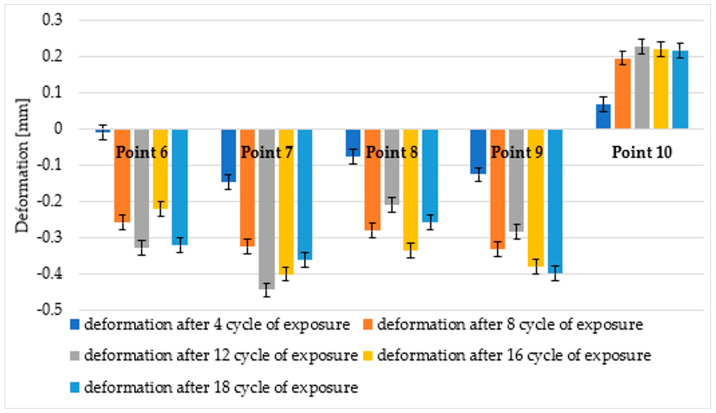
Deformation at the measurement points on the fluted surface during successive ageing stages. The error bars show the expanded measurement uncertainty estimated as described in Section 3.4.

The measurement inspection of the WPC composite profiles was performed using Gemoagic Control X (version: 2024.1.0. OQTON, INC, San Francisco CA 94104, USA)The results are presented as an example of a selected sample after two inspection steps, that is, after four and eight cycles of ageing in the form of a strain map on which the measuring points were additionally plotted (Figure 4 and Figure 5). However, point measurements of deformation after five consecutive inspections are summarised in Figure 6 for the brushed surface and in Figure 7 for the fluted surface analysed sample.

Detailed analysis of the statistical significance of differences between deformations is presented at point 3.3.

### 3.2. Microstructure Analysis

**Figure 8:**
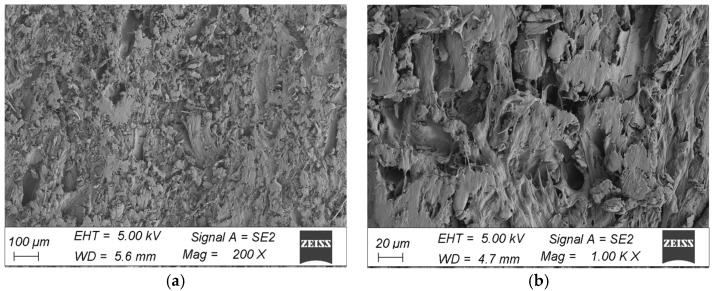
Microstructure of the surface of WPC profiles in the initial state (fragment taken from the corner of the profile), at magnification: (**a**) 200×, (**b**) 1000×.

**Figure 9:**
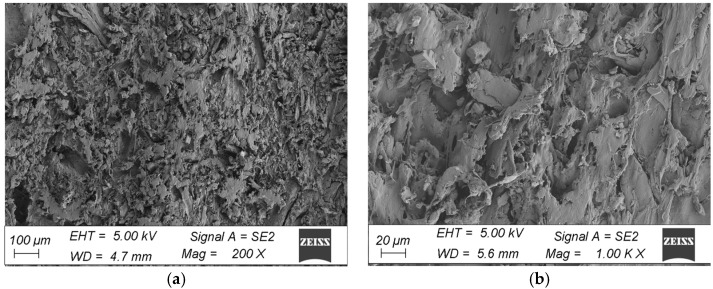
Microstructure of the surface of WPC profiles in the initial state (fragment taken from the centre of the profile), at magnification: (**a**) 200×, (**b**) 1000×.

**Figure 10:**
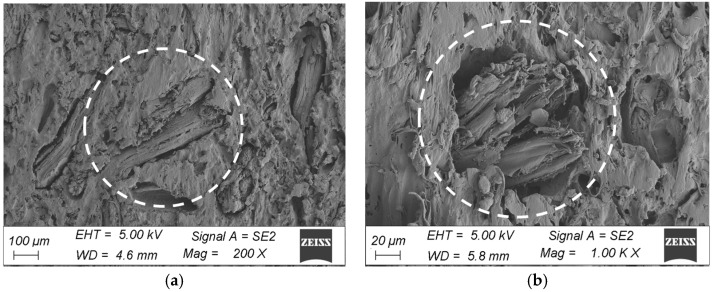
Microstructure of the surface of WPC profiles after ageing (fragment taken from the corner of the profile), at magnification: (**a**) 200×, (**b**) 1000×.

**Figure 11:**
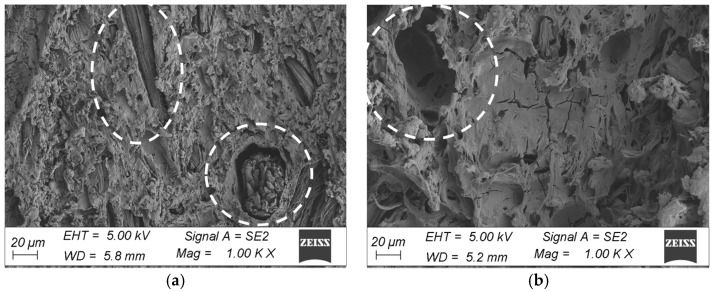
Microstructure of the surface of WPC profiles after ageing (fragment taken from the centre of the profile), at magnification: (**a**) 200×, (**b**) 1000×.

The morphological analysis consisted of a qualitative assessment of the surface and verification of damage to the surface layer of the profiles. A comparison was made between the surface taken from the corner of the profile and the fragment taken from the middle part of the profile in the initial state and after ageing. The microstructure of the surface of the WPC wood flour composite samples in the initial state indicated that the fibres were homogeneously coated with polymer, with no visible filler agglomerates or exposed wood fibres in the images. The surfaces of the profiles showed characteristically orientated streaks and scratches, indicative of the surface topography of the composite resulting from the brushing process (Figure 8 and Figure 9). In comparison, subjecting the composite profiles to an ageing process clearly changed the surface morphology. Microscopic images showed melting of the outer layer of the polymer, revealing surfaces of nonmoistened filler in the form of large lamellae and agglomerates of wood fibres, which were observed in composites taken from the centre of the profile and from the corners of the WPC profile. Changes in the morphology of the filler were also observed, that is, wood mace, where when the surface layer of the material was removed, the filler became visible in the form of interconnected fibres that formed characteristic clusters (Figure 10a and Figure 11a). In the composites taken from the central part of the profile, additional surface cracks were visible in the form of fissures, which deepened and widened in various places, forming a network of cracks and leading to degradation of the surface layer (Figure 11b). The aged samples from the central part of the profile also showed additional filler removal, which is visible in the images as oval hollow areas (Figure 11b).

### 3.3. Statistical Results

Despite the small number of samples in the result sets, the large number of sets subjected to Shapiro–Wilk tests allowed us to draw conclusions about the distributions of the results.

(P) In most sets (60%) in which the variable was a point in the sample, the null hypothesis that the set came from a normally distributed population was not confirmed.
(S) The sets in which the variable was a sample, the Shapiro–Wilk test confirmed the hypothesis that the sets came from a normally distributed population in 96% of the sets in which the variable was a sample.
(E) The Shapiro–Wilk test confirmed the hypothesis that the sets came from a normally distributed population in 90% of the sets, in which exposure time was a variable.

Based on the above results, an ANOVA analysis should be performed only for the sets in which the variable was a sample or exposure time because the normal distribution was not confirmed in the sets in which the variable was a point on the sample. However, to obtain a general picture of the situation, a two-way analysis of variance (ANOVA) was performed for all variables. The results of the analysis are presented in Table 2.

Considering that the influence of the sample on the results was statistically insignificant in most cases (Table 2a,b,c), the results obtained for all samples were averaged. Factors on which the deformation results may depend are assumed to be the point of the sample and the number of stages of ageing. Table 3 presents the differences between the deformations measured in the individual age stages separately for each point of the samples (1, 2, …, 10 markings according to Figure 4). The notation “x y cycles” means the difference between the average deformation for five samples at a given point after the y ageing cycles and after the x cycles. The differences for which statistical significance was confirmed are shown in the grey background.

### 3.4. Measurement Uncertainty

The measurement uncertainty was estimated from ten measurements taken at the same point (each time, the entire measurement procedure was repeated).

For each measurement, a result was obtained together with the standard deviation *s*_1_ assigned to the individual result estimated by the inspection software. This standard deviation results from the distribution of the surface point cloud of the compared area representing the measurement point. Because the images are three-dimensional, the measurement point is represented by a sphere with a corresponding diameter. In this study, a sphere with a diameter of 3 mm was used. All points inside this sphere were subjected to statistical analysis, for which the standard deviation was determined.

Based on the ten deviations *s*_1*i*_, *i* = 1, …, 10, the mean standard deviation for the point, *s*_1_, was obtained. *s*_1_ = 0.0078 mm.

The standard deviation *s*_2_ of the ten results obtained was taken as the standard deviation of repeatability. *s*_2_ = 0.0030 mm.

The standard uncertainty of the measurement of um was assumed to be:

(4) $$u_{m}=\sqrt{s_{1}^{2}+s_{2}^{2}}$$

The value of the standard uncertainty of the measurement obtained in this way is *u_m_* = 0.008 mm. Considering the coverage interval of 95%, the final result is an extended uncertainty of measurement of *U_m_* = 0.02 mm.

Table 4 shows the differences in deformation between the individual points of the samples after the subsequent stages of exposure. The numbers in the table indicate the difference in the sample deformation at the point x-listed horizontally in the header and y-listed vertically, after the subsequent stages of ageing. The differences between the points for which statistical significance was confirmed are shown in the grey background.

## 4. Discussion

Three-dimensional scanning enables the acquisition of detailed images of the test models. The accuracy of the obtained results depended on the scanning technique used. In this study, a metrology-grade laser scanner was used. The small size of the models and stable observation conditions allow for a highly accurate three-dimensional mapping of the surface of the test model. At least two scans were required to quantitatively and qualitatively assess changes in geometry. The first is a reference scan before the geometry changes, and the second is a model scan after the model deformation. A comparison of these scans was performed using dedicated inspection software. This task was performed in this study. The use of a high-accuracy measurement method does not guarantee that the results obtained in a test procedure involving this measurement will have high accuracy. The standard uncertainty of the measurement for the presented test method was estimated to be 0.008 mm, which, assuming a normal distribution and 95% coverage interval, gives an approximate expanded uncertainty value of 0.016 mm. Therefore, presenting the results with an accuracy of hundreds of millimetres is metrologically justified.

However, an essential feature of a test method is that after completion of the full test procedure, the results for internally different products or phenomena should differ from each other, which can be called the “resolution of the test method”. The test method used, which consists of ageing the composite profiles and then assessing their deformation using 3D scanning, was analysed to determine their “resolution”.

The results of the two-way ANOVA were not entirely conclusive (Table 1). However, the authors assumed that although the sets of results were few, the number of sets was relatively large. Based on the number of sets in which statistically significant differences were confirmed, it can be summarised as follows:

- Because in most cases (nine out of ten when the second influencing factor was the point in the sample and eight out of ten when the second influencing factor was exposure time) the differences between the results for individual samples were found to be statistically insignificant, it can be concluded that the deformation differences for individual samples are statistically insignificant. This means that the effect of the sample is insignificant; therefore, the set of results obtained from several samples can be regarded as a set in which the dispersion of results is random and the mean of the results for the individual samples can be regarded as the authoritative result. Confirmation of such conclusions can also be found in the Shapiro–Wilk tests, which, in sets where the sample was variable, confirmed the hypothesis of a normal distribution in 96% of cases.
- In all sets of results, a two-way analysis of variance (ANOVA) showed statistically significant differences in deformation at different points on the samples, both when the other influencing factors were the sample and the exposure time.
- For deformation differences according to the exposure time, the situation is not clear. Only half of the ANOVA results indicated that these differences were significant.

Although the two-way ANOVA was not fully conclusive, the one-way analysis of variance, comparing pairs of single sets of results, provides more detailed information (Table 2 and Table 3).

At all sample points, statistically significant changes in deformation occurred up to cycles four or eight. For the differences between the deformations found in successive ageing stages: after 12 cycles, after 16 cycles, and after 18 cycles, one-way analysis of variance did not confirm statistical significance at any point in the samples. Thus, using the measurement technique in question, it is possible to assess at what stage of ageing the most significant changes occur.

For all exposure times, the differences between the deformations of the points placed in the corners of the specimens: 1-2, 1-3, 1-4, 2-3, 2-4, and 3-4 (as indicated in Figure 4 and Figure 5) are insignificant, while the differences between the individual corner points 1, 2, 3, 4, and point 5 in the centre of the specimen are statistically significant. Similarly, on the fluted side of the sample, the deformation of the corner points (6, 7, 8, and 9) was not statistically significantly different, while the deformation of the centre point (10) was significantly different from that of the corner points. This can also be visually observed in the strain maps shown in Figure 4 and Figure 5. Therefore, statistical analysis confirms that the deformation measurement technique is suitable for determining the nature of deformation. In this case, it was the formation of a convexity in the central part of the samples. The nature of the deformation was the same for all samples.

Depending on the sets of results analysed, the smallest differences between deformation values of approximately 0.08 mm were assessed as statistically significant. The largest values assessed as nonsignificant did not exceed 0.17 mm. This provides information on the resolution of the method, which can be approximated at 0.2 mm.

As mentioned in Chapter 1 of this paper, the durability in relation to the geometry of composite decking is limited to swelling tests after exposure to moisture. The requirements for the change in linear dimensions under the influence of moisture (28 days) presented in the EAD for individual results are as follows:

- 5% in thickness, which for a thickness of 25 mm is <1.25 mm
- 1.2% in width, which for a width of 180 mm is 2.16 mm.

The actual swelling results in the thickness of composite boards with a PVC matrix and a filler of fine lignocellulosic fibres being generally in the range of 0.2 to 0.5 mm.

In the case in question, the out-of-plane deformations under ageing were 0.15 to 0.42 (Table 3) at the individual points, and the average differences in the deformations between the points reached 0.7 mm (Table 4). Therefore, out-of-plane deformation may be noteworthy in terms of the durability of decking boards and is comparable to the changes caused by swelling under the influence of moisture.

3D scanning, unlike other methods of measuring deviation from flatness (e.g., using a feeler gauge or bow gauge for measuring cupping shown in EN 15534-1 [31], allows for capturing the nature of the sample deformation. Statistical analysis showed that the measurement method provided the ability to determine the effect of various ageing stages and to determine the nature of the deformation based on the deformation at various points in the sample.

The subjecting of the composite profiles to an ageing process clearly changes the surface morphology. Microscopic images highlighted the melting of the outer layer of the polymer, revealing the surfaces of the unweathered filler in the form of large lamellae and agglomerates of wood fibres, which can be observed in the composites after ageing for fragments coming from both the corner and middle areas of the profile (Figure 10a and Figure 11a). Changes in filler were also observed, swelling, and visible in the form of clusters [32], which is particularly evident for fragments taken from the corner of the profile (Figure 10b). Microstructural analysis showed a change in the nature of the failure of the surface layer of the profile, depending on the location of the material used for testing. The sample taken from the corner of the profile was less degraded compared to the fragments coming from the centre of the profile. In the case of the corners, the filler is exposed, detached from the matrix, and swells (Figure 10a,b). In the ageing specimens taken from the central part of the profile, additional filler salvage occurred, which can be seen in the images as oval hollow areas. Furthermore, the polymer matrix itself changes with cracking of the surface layer [33] of polyvinyl chloride (Figure 11a,b), which is particularly evident at higher magnifications (Figure 11b).

Microstructural analysis of the samples after ageing showed that exposure of the profiles to light emitted by fluorescent lamps, alternating with humidification, led to significant surface degradation, depending on the nature of the damage, depending on the place the material was taken for testing. The greatest changes in the surface of the profile were observed in the sample obtained from the central part of the profile. In the morphology analysis, melting of the top layer of the polymer matrix [34], exposure of the wood fibre surface [35], fracturing of the top layer of polyvinyl chloride, and loss of filler from the polymer matrix became apparent [36]. In the 3D scan inspection tests, relatively large deformations were also observed in the central part of the sample, which means that this area was subjected to strong geometrical changes during environmental impacts.

The results of the SEM observations confirmed the differences that could also be observed in the deformation measurements. The largest deformations detected by the inspection programme indicate, at the same time, areas of marked changes in the structure of the material.

## 5. Conclusions

Both the estimated measurement uncertainty and the statistical evaluation of the resolution of the test method show that it is reasonable to conclude that the 3D scanning method used allows the differences between the deformations of the composite samples to be assessed at a level not worse than 0.2 mm. On the basis of the comparison with the requirements for swelling (change in linear dimensions) of composite decking boards under the influence of moisture, it was concluded that with a similar level of requirements for out-of-plane deformation under ageing, the method would be metrologically suitable for its evaluation. The method allows conclusions to be drawn about the deformation occurring in the samples under the influence of ageing and the differences in deformation at individual locations and after successive stages of ageing. The 3D scanning method, in combination with inspection analysis, has proven to be effective in detecting micro-deformations in zones that have not yet been considered during standard measurements. This is particularly important in combination with changes in the microstructure of the material. This is because it turns out that the largest deformations detected by the inspection programme indicate areas of marked changes in the structure of the material at the same time. Thus, including an out-of-plane deformation assessment after environmental impacts may be important in assessing the durability of composite decking for outdoor applications. The 3D scanning method is a suitable and convenient method for this purpose.

In the future, the research team plans to measure deformations in larger elements, such as a fragment of a wall covered with WPC composite panels. Large-area elements will be exposed to humidity and thermal factors. This research will contribute to the method developed for the diagnostics of composite facade cladding using 3D scanning.

## Figures and Tables

**Table 1:** Ageing exposure.

Total Duration of Exposure	Total Number of Cycles	Exposure Pattern Over the Cycle
3024	18	24 h of humidification by condensation at temperature 45 ± 3 °C 168 h of alternating irradiation and sprinkling, in the sequence: (a) 2.5 h of irradiation with UVA-340 lamps, irradiance 0.89 watt per square metre (340 nm), temperature (Black Standard Thermometer) 60 ± 3 °C, (b) 0.5 h of water sprinkling, no UV, sprinkling rate 6–7 litre per minute.

**Table 2:** Results of the two-way ANOVA analysis determining the effect of the sample, the sample point and exposure time on sample deformation. (a) Effect of the sample and exposure time tested for each point separately, (b) effect of the sample point and exposure time tested for each sample separately, and (c) effect of the sample and sample point tested for each exposure time separately. SSD: statistically significant differences (influence of the factor is significant).

**(a)**					
	**Sample Point Number**				
**Influences**	**1**	**2**	**3**	**4**	**5**
Effect of the sample	SSD	SSD			
Effect of the time of exposure	SSD	SSD	SSD		SSD
	**Sample Point Number**				
**Influences**	**6**	**7**	**8**	**9**	**10**
Effect of the sample					
Effect of the time of exposure		SSD		SSD	
**(b)**					
	**Sample**				
**Influences**	**A**	**B**	**C**	**D**	**E**
Effect of the point on the sample (points 1–5)	SSD	SSD	SSD	SSD	SSD
Effect of exposure time of exposure (points 1–5)			SSD		
Effect of the point on the sample (points 6–10)	SSD	SSD	SSD	SSD	SSD
Effect of exposure time of exposure (points 6–10)	SSD			SSD	SSD
**(c)**					
	**Time of Exposure-Number of Cycles**				
**Influences**	**4 Cycles**	**8 Cycles**	**12 Cycles**	**16 Cycles**	**18 Cycles**
Effect of the Sample (points 1–5)			SSD		
Effect of the point on the sample (points 1–5)	SSD	SSD	SSD	SSD	SSD
Effect of the sample (points 6–10)					
Effect of the point on the sample (points 6–10)	SSD	SSD	SSD	SSD	SSD

**Table 3:** Differences between mean deformations (five samples) in subsequent ageing stages at individual sample points.

The Point on the Sample	Mean Deformation After 4 Cycles	4÷ 8 Cycles	8 ÷ 12 Cycles	12÷ 16 Cycles	16÷ 18 Cycles	8 ÷ 18 Cycles	Total Mean Deformation After 18 Cycles
	Deformation Differences, mm						
Point 1	0.146	0.177	0.035	−0.042	0.104	0.097	**0.42**
Point 2	0.179	0.145	0.007	0.023	0.058	0.088	**0.41**
Point 3	0.251	0.142	0.059	−0.078	0.045	0.026	**0.42**
Point 4	0.223	0.167	0.006	−0.074	0.063	−0.005	**0.38**
Point 5	−0.082	−0.166	−0.009	0.015	0.020	0.027	**−** **0.22**
Point 6	−0.175	−0.208	0.002	−0.031	0.040	0.011	**−** **0.37**
Point 7	−0.178	−0.189	−0.088	0.111	−0.051	−0.028	**−** **0.39**
Point 8	−0.102	−0.278	0.022	−0.015	0.089	0.097	**−** **0.28**
Point 9	−0.159	−0.208	−0.026	0.041	−0.030	−0.015	**−** **0.38**
Point 10	0.139	0.0780	0.033	−0.051	−0.049	−0.066	**0.15**

**Table 4:** Differences between deformations of individual points in the samples.

**Exposure**		**Point 1**	**Point 2**	**Point 3**	**Point 4**
	**Deformation Differences, mm**				
4 cycles	Point 2	−0.032	-	-	-
	Point 3	−0.105	−0.073	-	-
	Point 4	−0.076	−0.044	0.029	-
	Point 5	−0.229	0.261	0.334	0.305
8 cycles	Point 2	−0.001	-	-	-
	Point 3	−0.070	−0.069	-	-
	Point 4	−0.066	−0.065	0.004	-
	Point 5	0.571	0.572	0.642	0.638
12 cycles	Point 2	0.028	-	-	-
	Point 3	−0.094	−0.121	-	-
	Point 4	−0.037	−0.065	0.057	-
	Point 5	0.616	0.588	0.709	0.652
16 cycles	Point 2	−0.038	-	-	-
	Point 3	−0.058	−0.020	-	-
	Point 4	−0.005	0.033	0.053	-
	Point 5	0.558	0.596	0.616	0.563
18 cycles	Point 2	0.009	-	-	-
	Point 3	0.001	−0.008	-	-
	Point 4	0.036	0.028	0.036	-
	Point 5	0.642	0.634	0.642	0.606
**Exposure**		**Point 6**	**Point 7**	**Point 8**	**Point 9**
	**Difference, mm**				
4 cycles	Point 7	0.002	-	-	-
	Point 8	−0.073	−0.075	-	-
	Point 9	−0.017	−0.019	0.056	-
	Point 10	−0.315	−0.317	−0.241	−0.298
8 cycles	Point 7	0.017	-	-	-
	Point 8	0.003	−0.014	-	-
	Point 9	0.017	0.000	0.014	-
	Point 10	0.601	0.584	0.598	0.584
12 cycles	Point 7	−0.073	-	-	-
	Point 8	0.023	0.096	-	-
	Point 9	−0.011	0.062	−0.034	-
	Point 10	0.632	0.705	0.609	0.643
16 cycles	Point 7	0.069	-	-	-
	Point 8	0.039	−0.030	-	-
	Point 9	0.061	−0.008	0.022	-
	Point 10	0.612	0.543	0.573	0.551
18 cycles	Point 7	−0.022	-	-	-
	Point 8	0.089	0.111	-	-
	Point 9	−0.009	0.014	−0.097	-
	Point 10	0.523	0.546	0.435	0.532

## Data Availability

The data presented in this study are available on request from the corresponding author (for privacy reasons).
